# Supplementary figures and images for: Phototrophy and carbon fixation in Chlorobi postdate the rise of oxygen
Source: PLoS One. 2022 Aug 1;17(8):e0270187. doi: 10.1371/journal.pone.0270187 (PMC9342728; doi:10.1371/journal.pone.0270187)

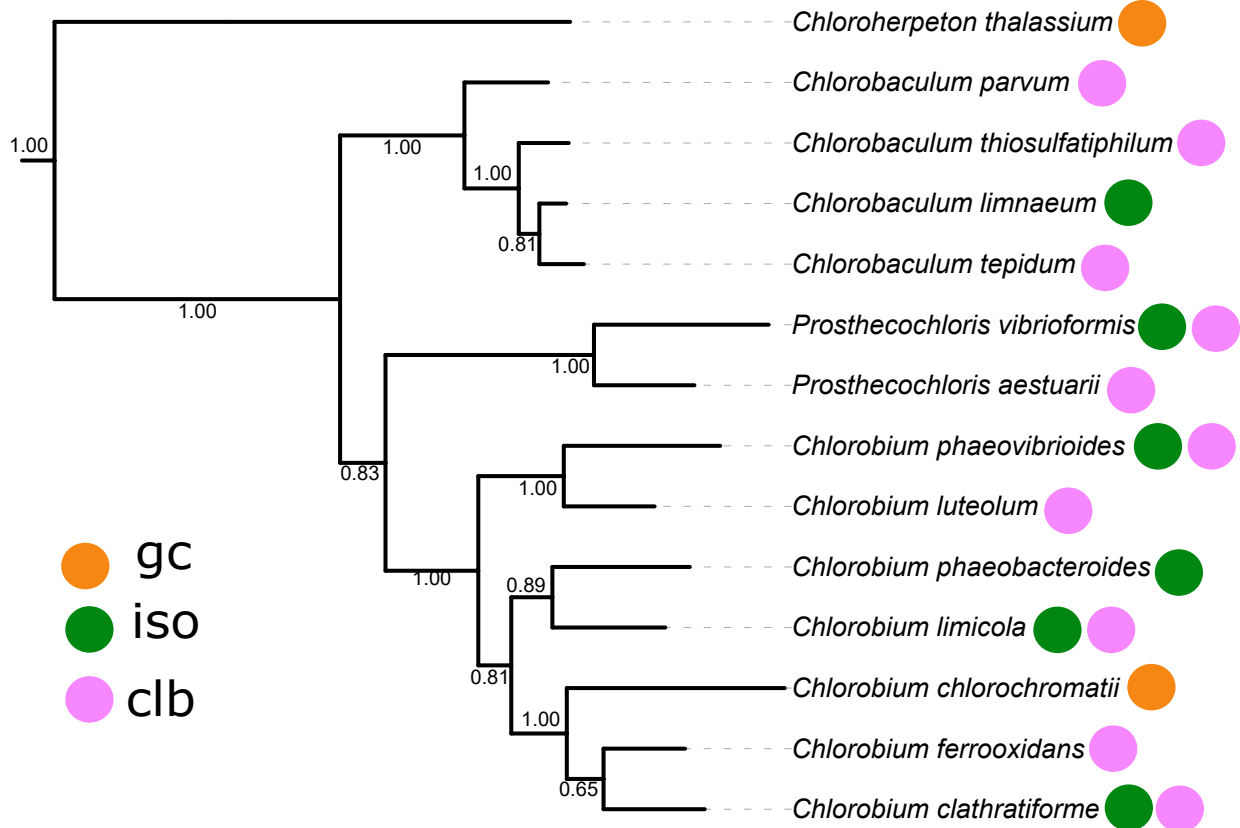

Tree scale: 0.1

Supplement: S1 Fig — (PDF) [file pone.0270187.s003.pdf]

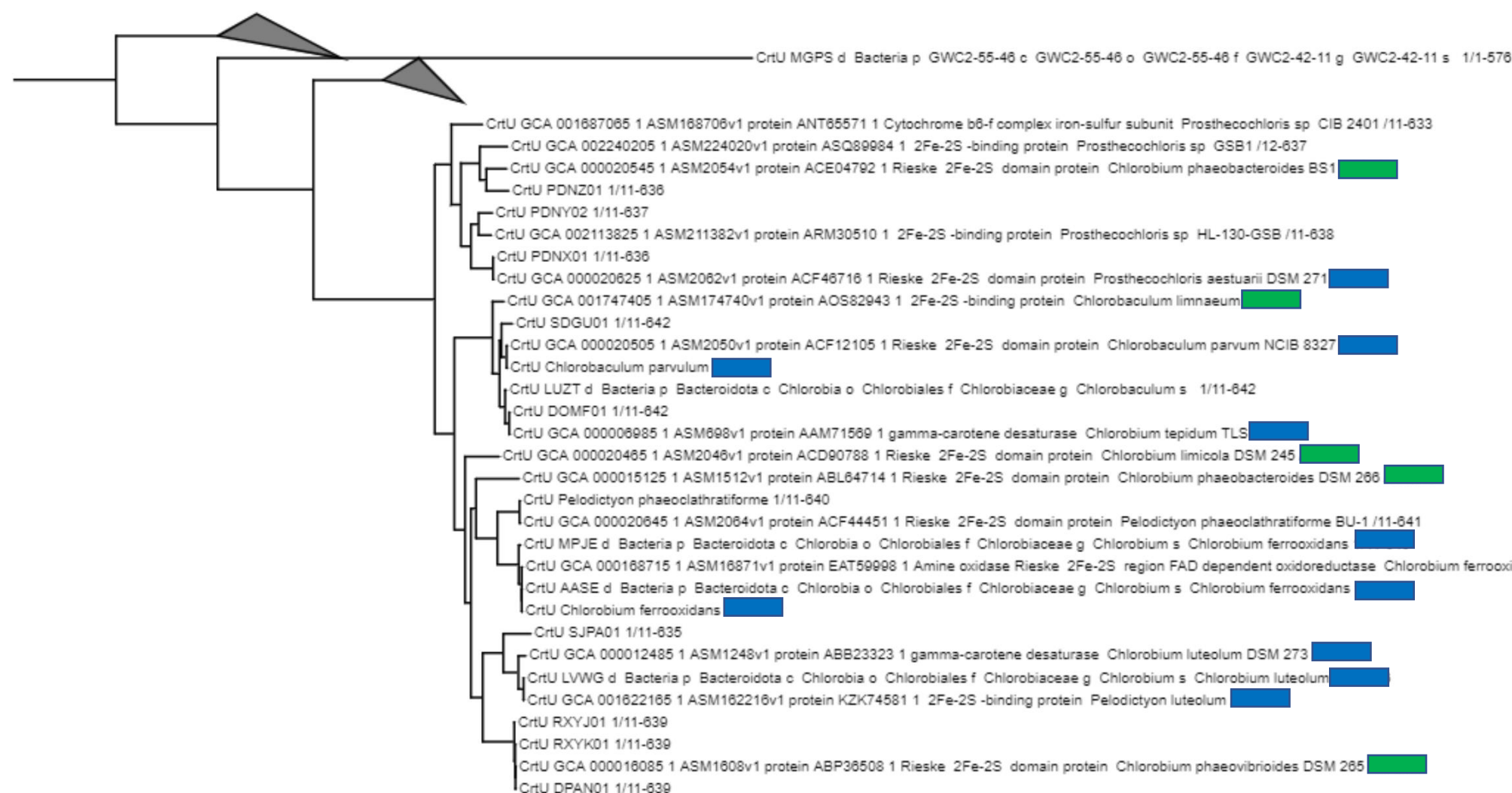

Gc  
Iso  
cb

Supplement: S2 Fig — (PDF) [file pone.0270187.s004.pdf]

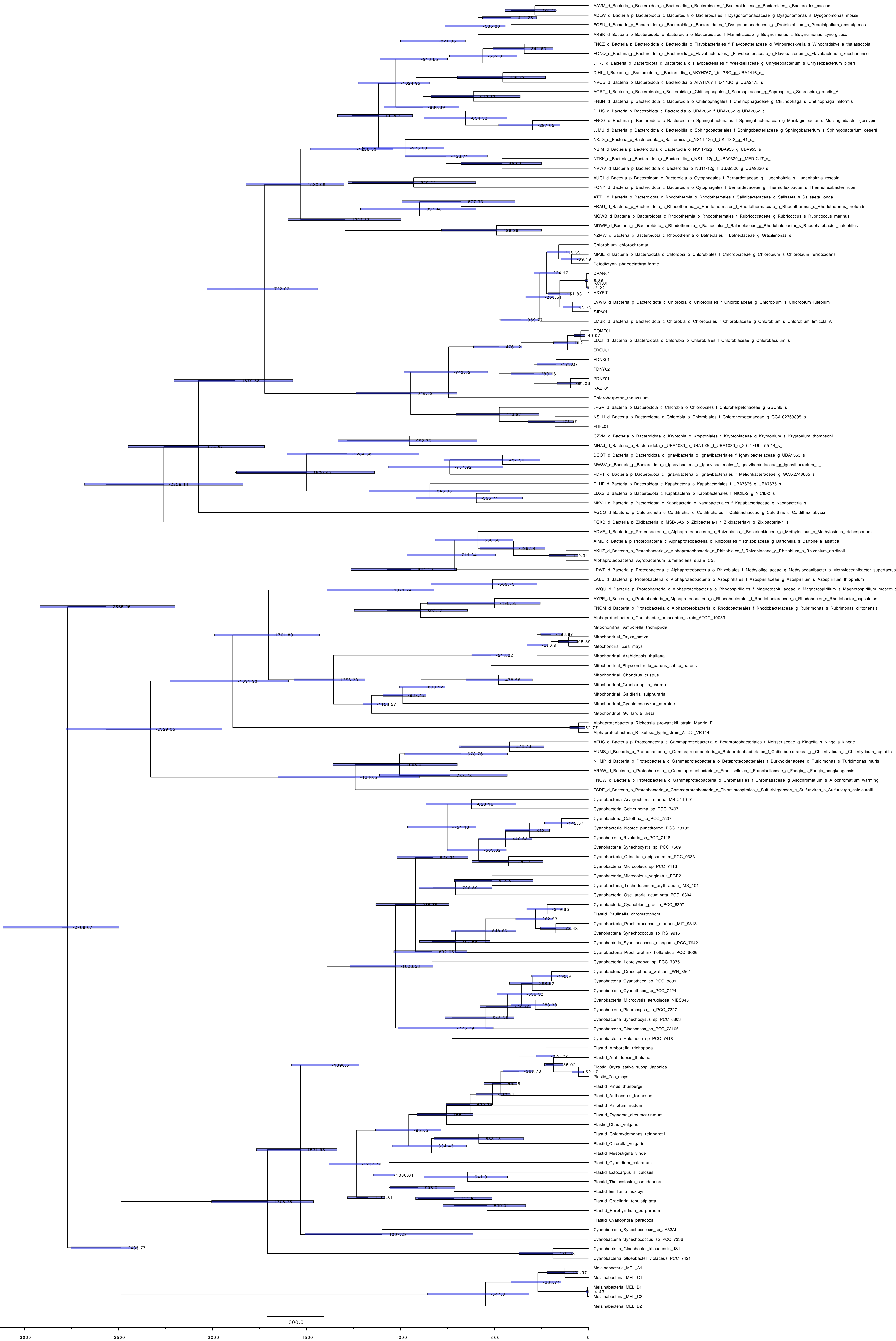

Supplement: S3 Fig — Cyanobacteria/Melainabacteria divergence constrained to a uniform prior 2400–3800 Mya. (PDF) [file pone.0270187.s005.pdf]

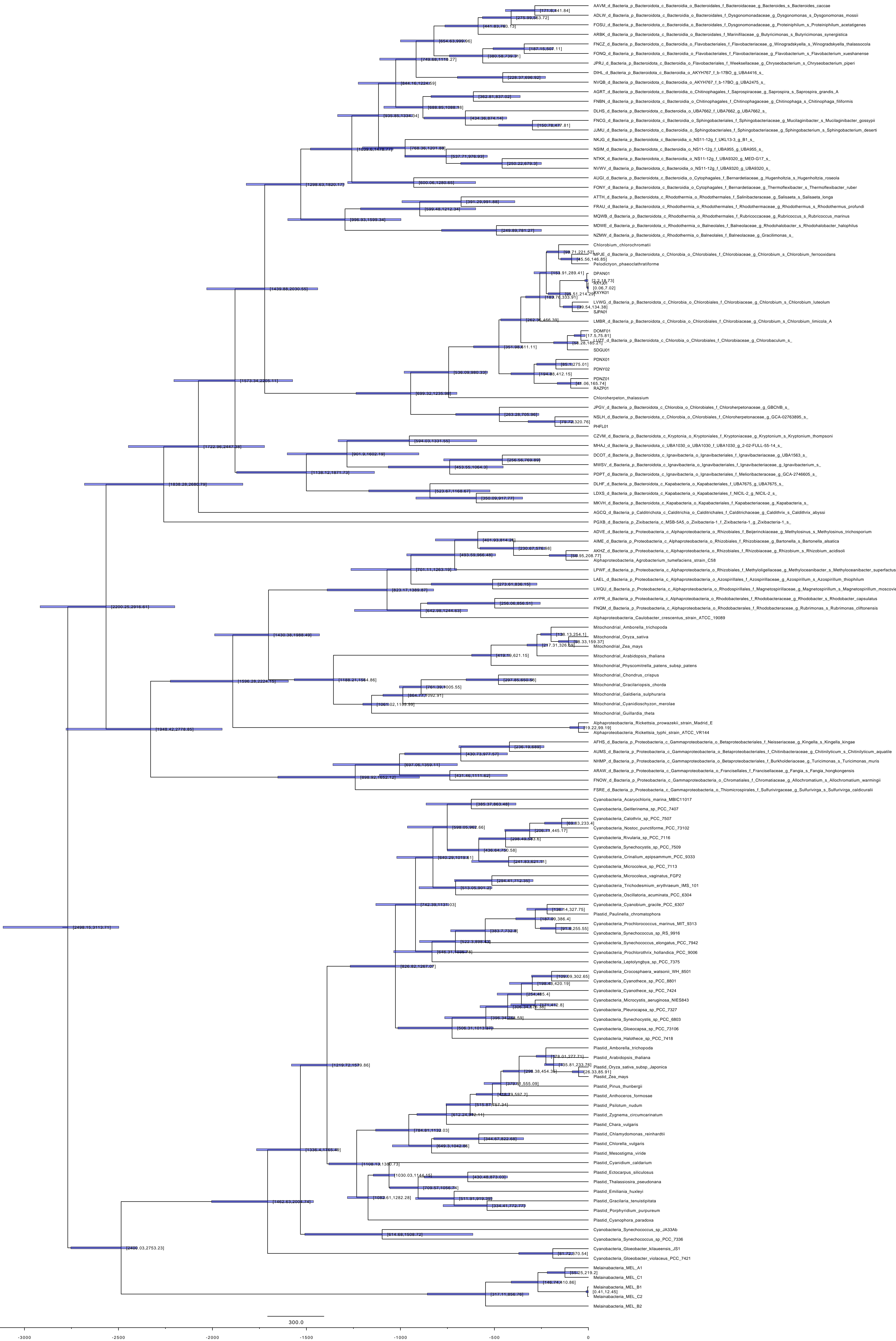

Supplement: S4 Fig — Cyanobacteria/Melainabacteria divergence constrained to a uniform prior 2400–3800 Mya. (PDF) [file pone.0270187.s006.pdf]

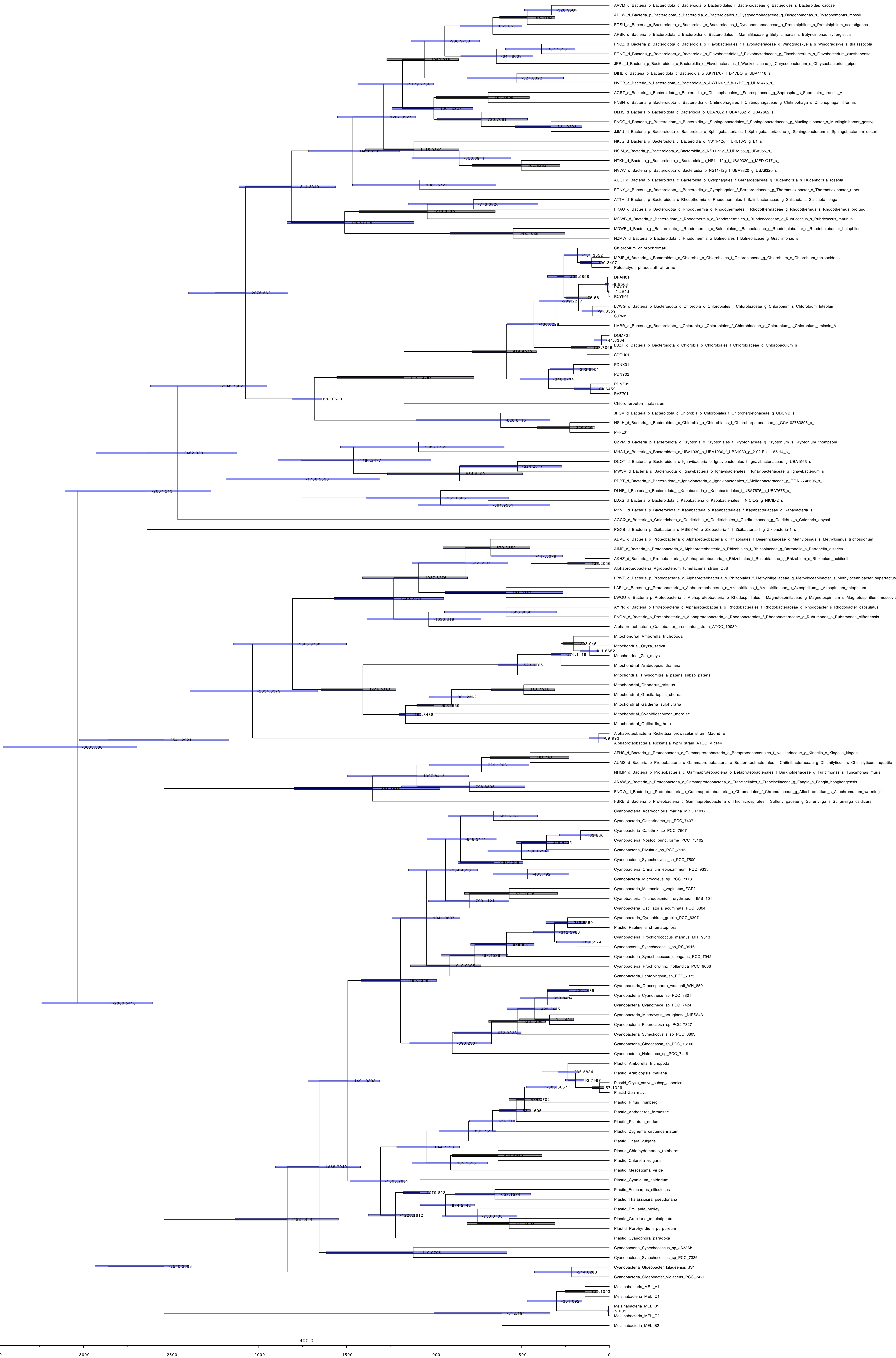

Supplement: S5 Fig — Most recent common ancestor of Chlorobi constrained to uniform prior between 1637–1643 Mya. Cyanobacteria/Melainabacteria divergence constrained to a uniform prior 2400–3800 Mya. (PDF) [file pone.0270187.s007.pdf]

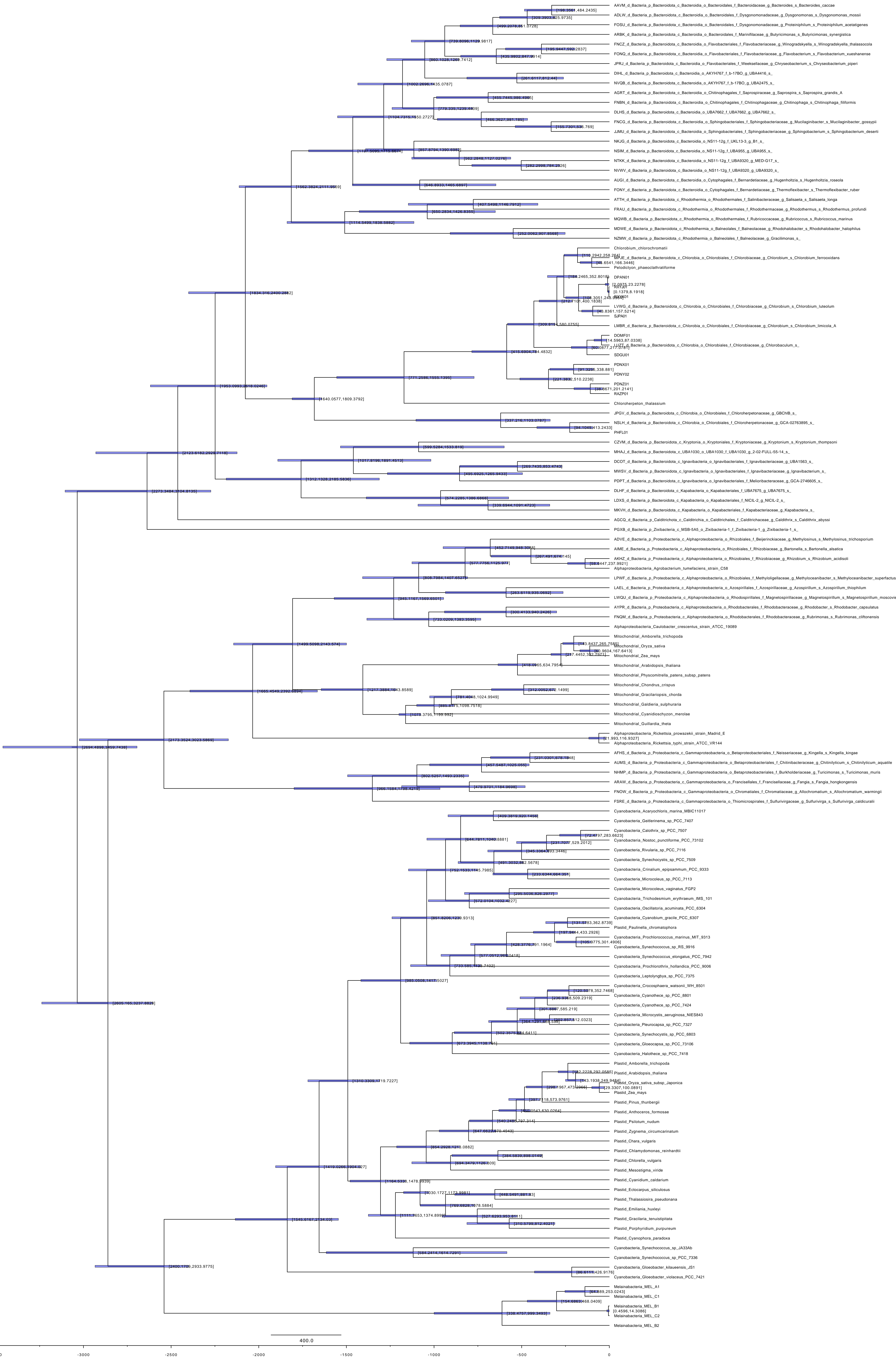

Supplement: S6 Fig — Most recent common ancestor of Chlorobi constrained to uniform prior between 1637–1643 Mya. Cyanobacteria/Melainabacteria divergence constrained to a uniform prior 2400–3800 Mya. (PDF) [file pone.0270187.s008.pdf]

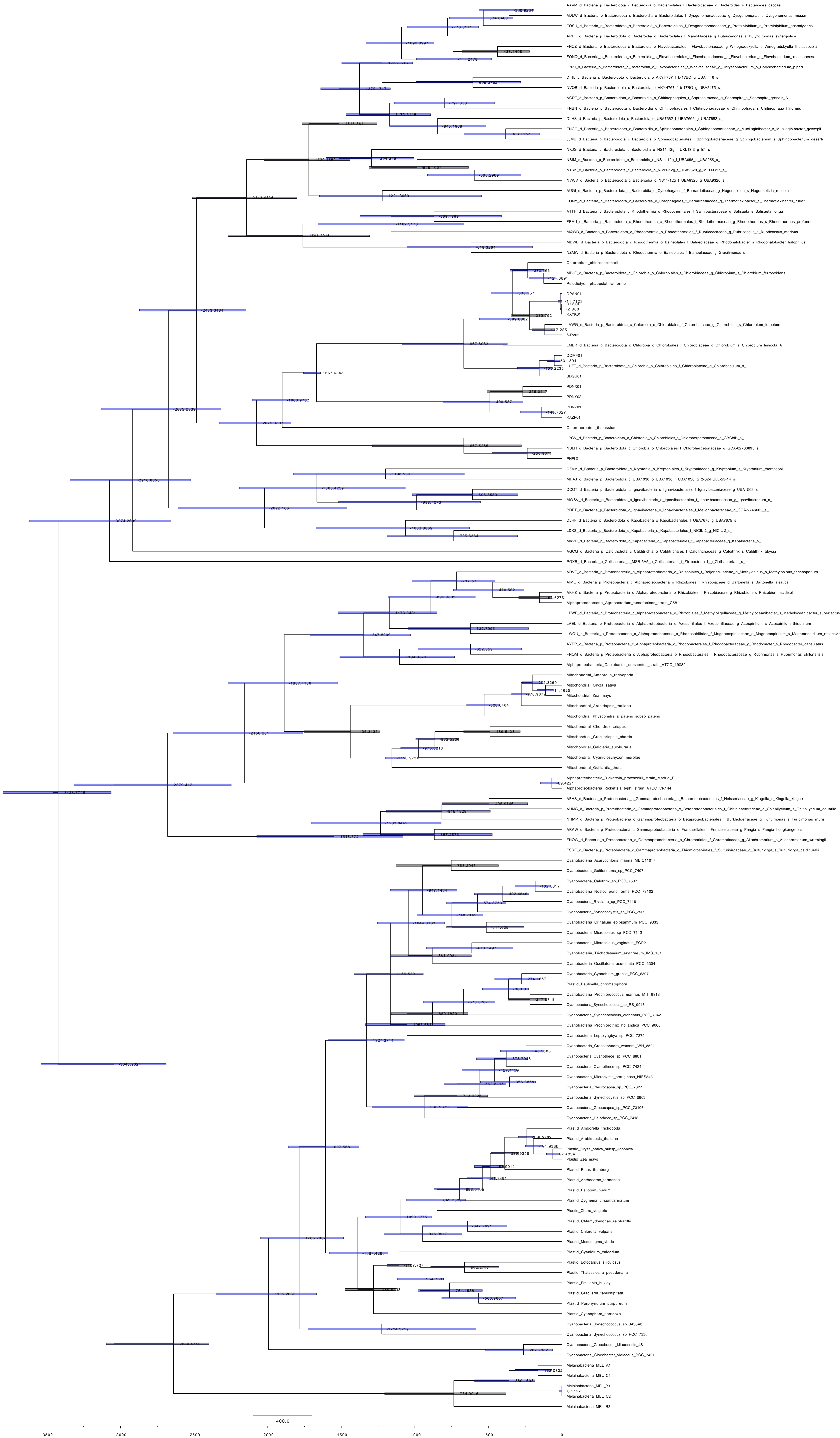

Supplement: S7 Fig — Most recent common ancestor of Chlorobiaceae constrained to uniform prior between 1637–1643 Mya. Cyanobacteria/Melainabacteria divergence constrained to a uniform prior 2400–3800 Mya. (PDF) [file pone.0270187.s009.pdf]

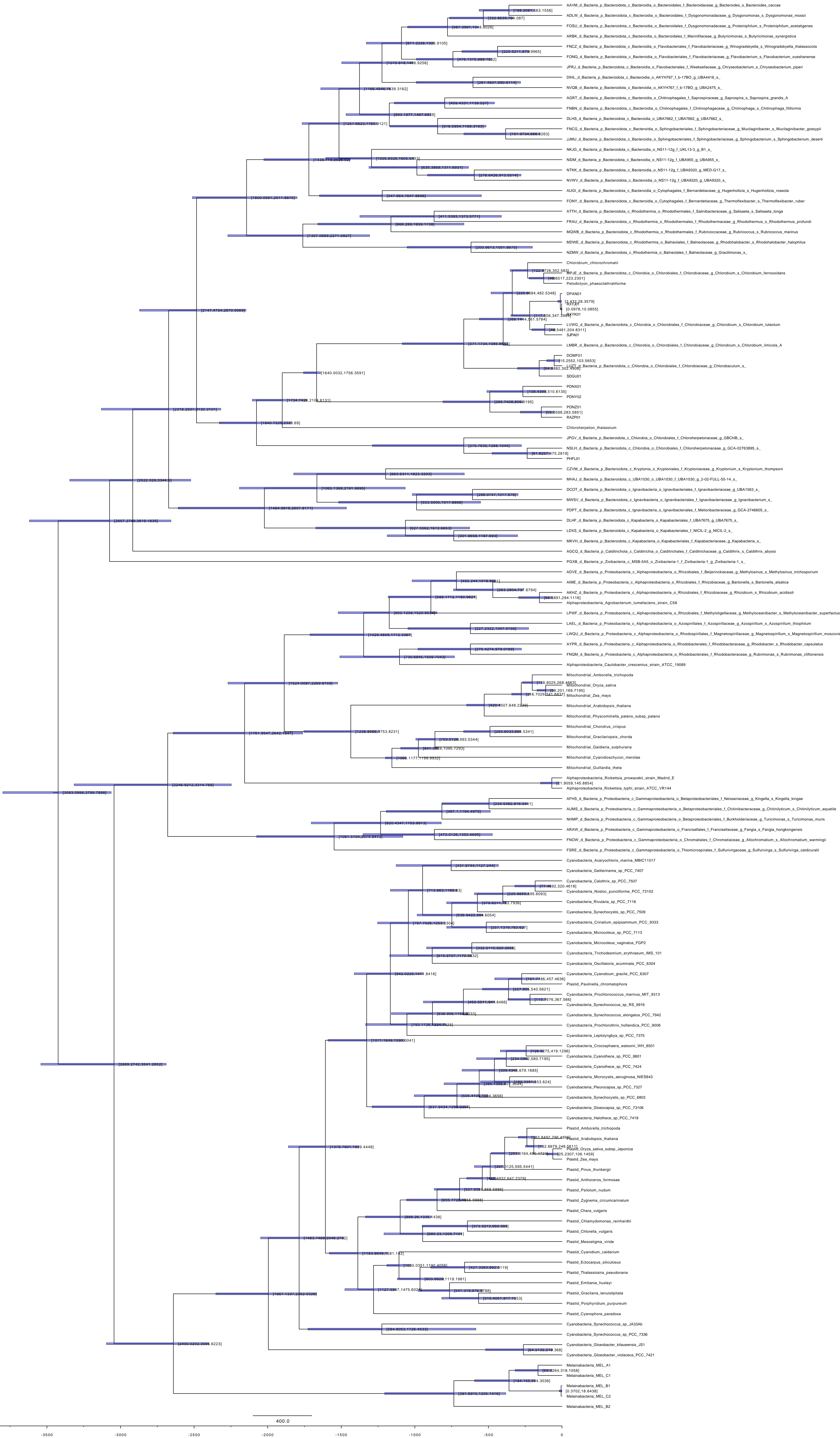

Supplement: S8 Fig — Most recent common ancestor of Chlorobiaceae constrained to uniform prior between 1637–1643 Mya. Cyanobacteria/Melainabacteria divergence constrained to a uniform prior 2400–3800 Mya. (PDF) [file pone.0270187.s010.pdf]
